# OTUD4 Inhibits Prostate Cancer by Deubiquitinating MYH9

**DOI:** 10.32604/or.2025.073455

**Published:** 2026-03-23

**Authors:** Zheng Qin, Yueyao Zhang, Dongze Liu, Xiaokang Zheng, Kaibin Wang, Xiao Zhu, Yuanhao Zhang, Kexin Xu, Changying Li, Lijuan Kang, Lili Wang, Haitao Wang

**Affiliations:** 1 Tianjin Key Laboratory of Precision Medicine for Sex Hormones and Diseases, The Second Hospital of Tianjin Medical University, Tianjin, 300211, China; 2 Department of Oncology, The Second Hospital of Tianjin Medical University, Tianjin, 300211, China; 3 Department of Cell Biology, Peking University Cancer Hospital & Institute, Beijing, 100142, China; 4 Department of Urology, The 2nd Affiliated Hospital of Harbin Medical University, Harbin, 150001, China; 5 Department of Urology, The Henan Provincial People’s Hospital, Zhengzhou, 462000, China

**Keywords:** Prostate cancer, ovarian tumor family deubiquitinase 4 (OTUD4), therapeutic target, myosin-9 (MYH9), ubiquitin (UB)

## Abstract

**Objective:**

Prostate cancer is the second most common fatal cancer in men. Identifying new biological therapeutic targets is crucial to effectively improve the prognosis of prostate cancer patients. Ovarian tumor family deubiquitinase 4 (OTUD4) is a member of the ovarian tumor-associated protease domain (OTUDs) family. Although previous studies have shown that the expression and function of OTUD4 vary across different tumors, its role in prostate cancer remains unknown. The aim of this study is to explore new therapeutic targets and diagnostic markers for prostate cancer and investigate their mechanisms of action.

**Methods:**

Cell culture, Cell Counting Kit-8 (CCK-8) assay, colony formation assay, Transwell assay, 5-Ethynyl-2^′^-deoxyuridine (EdU) assay, immunofluorescence, Western blot, Quantitative real-time PCR (qRT-PCR), protein mass spectrometry, nude mouse xenograft models, immunohistochemistry (IHC), and hematoxylin and eosin (H&E) staining were utilized.

**Results:**

We found that OTUD4 expression was reduced in prostate cancer and negatively correlated with poor prognosis in both *in vivo* and *in vitro* experiments. Subsequent mechanistic studies revealed that OTUD4 directly inhibits the degradation of myosin-9 (MYH9) protein via deubiquitination. Although MYH9 has been previously reported to act as a tumor suppressor in prostate cancer, no experimental evidence had demonstrated that MYH9 inhibits prostate cancer growth. Our results indicate that MYH9 overexpression effectively suppresses prostate cancer through interactions with cell adhesion molecules.

**Conclusion:**

Collectively, these results suggest that OTUD4 functions as a tumor suppressor in prostate cancer. Specifically, OTUD4 inhibits MYH9 degradation via deubiquitination, thereby enabling MYH9-mediated suppression of prostate cancer.

## 1 Introduction

Prostate cancer has become the second cancer leading cause of cancer death and places a heavy burden on global healthcare [1]. According to cancer statistics for 2023, the incidence of prostate cancer is rising by approximately 3% annually, equivalent to about 99,000 new cases per year [2]. Importantly, the growth of prostate cancer depends on androgens in the male body; therefore, androgen deprivation therapy (ADT) remains the cornerstone of early prostate cancer treatment. ADT can inhibit prostate cancer growth and delay clinical tumor progression [3]. However, after an average of two years of ADT, most patients inevitably progress to castration-resistant prostate cancer (CRPC), a condition in which prostate cancer continues to grow in a low-androgen environment and becomes unresponsive to ADT [4]. There is clear evidence that the use of steroid hormone drugs (Docetaxel, Cabazitaxel), androgen receptor targeted drugs (Enzalutamide, Apalutamide, Abiraterone) and radioactive metabolic therapy in the treatment of metastatic prostate cancer can effectively improve patient survival [5]. Enzalutamide is the most commonly used drug approved by the U.S. Food and Drug Administration (FDA) for the treatment of non-metastatic CRPC (nmCRPC) and metastatic CRPC (mCRPC). Unfortunately, most patients receiving enzalutamide develop resistance through a series of complex mechanisms, significantly reducing the drug’s efficacy [5]. Given the considerable limitations of current treatments, it is crucial to identify new biological therapeutic targets to effectively improve the prognosis of prostate cancer patients.

Protein metabolism is a fundamental biological process essential for normal cellular function, encompassing both protein synthesis and degradation. In eukaryotic cells, protein degradation occurs primarily through three systems: the mitochondrial protease pathway, responsible for degrading most mitochondrial proteins; the lysosomal pathway, which degrades cell membrane and endocytosed proteins; and the ubiquitin–proteasome system (UPS), which degrades various normal and abnormal intracellular proteins. Generally, most intracellular proteins are degraded via the UPS, making it the primary pathway for intracellular protein degradation. The ubiquitination process involves a series of ATP-dependent enzymatic reactions: ubiquitin (UB) is first activated by an activating enzyme (E1), then transferred to a cysteine residue on a conjugating enzyme (E2), and finally attached to the target protein by a ligase (E3), thereby completing ubiquitination and targeting the protein for degradation [6]. The ubiquitin–proteasome pathway maintains protein stability and homeostasis and participates in regulating numerous cellular processes, including autophagy, DNA damage signaling, inflammation, and other biological functions [7]. Ubiquitin ligases and deubiquitinases bind to substrates to regulate the ubiquitination and degradation of substrate proteins. Therefore, deubiquitination may regulate various cellular biological functions—such as inflammation, cell cycle arrest, tumor cell invasiveness, and cell death—by interacting with different substrate proteins [8,9].

Ovarian tumor family deubiquitinase 4 (OTUD4) is a deubiquitinase belonging to the ovarian tumor-associated protease domain (OTUD) family. Studies have shown that OTUD4 expression is significantly downregulated in 11 human cancers and is correlated with overall survival in patients with various tumors. An earlier study examining OTUD4 expression across multiple tumors revealed that OTUD4 mRNA levels were decreased in bladder urothelial carcinoma, breast invasive carcinoma, liver hepatocellular carcinoma, lung adenocarcinoma, and prostate adenocarcinoma [10]. In contrast, analysis of The Cancer Genome Atlas (TCGA) dataset indicated that OTUD4 expression was elevated in pancreatic adenocarcinoma, stomach adenocarcinoma, and thymoma [10]. Previous research on the role of OTUD4 in glioblastoma demonstrated that OTUD4 promotes glioblastoma progression by deubiquitinating CDK1 and activating the Mitogen-Activated Protein Kinase (MAPK) signaling pathway [11]. OTUD4 has also been shown to inhibit apoptosis in airway epithelial cells [12]. Similarly, OTUD4 was reported to promote pancreatic cancer progression by regulating glycolysis [13]. Conversely, a study on non-small cell lung cancer (NSCLC) found that OTUD4 suppresses NSCLC progression and enhances radiosensitivity by regulating the ATM/CHK2/P53 signaling pathway [14]. Thus, OTUD4 appears to be a key regulatory gene in tumor progression, primarily functioning through deubiquitination, although its role varies across different cancers. While no specific experiments have yet confirmed the function of OTUD4 in prostate cancer, it is known that OTUD4 expression is decreased in prostate adenocarcinoma. These findings sparked our interest.

Myosin consists of three domains: head, neck, and tail. The myosin family includes 12 different types [15]. The head regions of these myosins are highly conserved, while the tail domains confer functional specificity. Myosin II contains a tail composed of multiple coiled-coil regions, enabling it to perform contractile functions [16]. Both myosin II and non-muscle myosin II (NMII) belong to the myosin II family and are major motor proteins [17,18]. NMII includes three subtypes: NMIIA, NMIIB, and NMIIC [19]. The myosin heavy chain 9 (MYH9) gene, located on chromosome 22q12.3, encodes the heavy chain of NMIIA. Mutations in MYH9 are associated with several diseases, such as Sebastian platelet syndrome, Fechtner syndrome, and Epstein syndrome. Importantly, recent studies have revealed a significant link between MYH9 and tumor development [20]. Initially identified as a tumor suppressor, MYH9 interacts with multiple signaling pathways and key molecules, including AKT Strain Transforming (AKT1), p53, Myelocytomatosis Oncogene (Myc), Epidermal Growth Factor Receptor (EGFR), MAPK1, and Jun, to influence tumorigenesis [20]. Earlier studies suggested that MYH9 inhibits the development of squamous cell carcinoma and melanoma [21,22]. However, subsequent research indicates that MYH9 plays a dual role in head and neck squamous cell carcinoma, suppressing tumor initiation and progression while also being associated with tumor invasion and radioresistance [23,24]. To date, no studies have elucidated the role of MYH9 in prostate cancer.

Therefore, this study aims to investigate the impact of OTUD4 on prostate cancer and explore its underlying mechanisms. Another aim of this study is to explore the function of MYH9 in prostate cancer and identify downstream signaling pathways that MYH9 may affect.

## 2 Methods

### 2.1 Cell Culture

The C4-2 (Procell, CL-0182, Wuhan, China) and PC-3(Procell, CL-0185, Wuhan, China) were cultured with RPMI 1640 medium (BasalMedia, L210KJ, Shanghai, China) mixing 10% fetal bovine serum (FBS) (HAKATA, HB-FBS-500, Shanghai, China), 1% penicillin and streptomycin (P/S) (BasalMedia, S110JV, Shanghai, China) at 37°C with 5% CO_2_. All the cell lines used in this study were verified to be free of mycoplasma contamination and authenticated through short tandem repeat (STR) profiling.

### 2.2 Chemicals and Antibodies

Cell Counting Kit-8 (CCK-8) (APExBIO, K1018, Houston, TX, USA) were acquired in APExBIO. RIPA (Solarbio, R0020, Beijing, China), PMSF (Solarbio, IKM1104), Paraformaldehyde (Solarbio, P1110) and Crystal Violet Desalination Solution (Solarbio, G1064) were purchased from Solarbio. The enhanced chemiluminescence (ECL) detection reagent (PK10003) was purchased from Proteintech (Wuhan, China). TRIzol reagent (99089501) was purchased from Ambion (Thermo Fisher Scientific, Austin, TX, USA). Anti-OTUD4 (25070-1-AP), anti-GAPDH (60004-1-Ig), anti-Beta Actin (66009-1-Ig), anti-MYH9 (81204-1-RR), anti-N-Cadherin (22018-1-AP), anti-E-Cadherin (20874-1-AP), anti-MMP9 (10375-2-AP), anti-Flag (20543-1-AP), anti-His (66005-1-Ig), anti-Vimentin (10366-1-AP), anti-Ki-67 (27309-1-AP) and anti-Phospho-Histone H2A.X (Ser139)(83307-2-RR) were obtained from Proteintech.

### 2.3 Western Blot Assay

The original medium was discarded, washed twice with PBS, then RIPA and PMSF (100:1) were mixed, and the cells were lysed at 4°C for 60 min. The solution was collected and centrifuged at 11,000× *g* for 15 min to obtain the protein supernatant at 4°C. The Bradford method was used to determine protein concentration. The volume of a 30 μg sample was calculated from the measured concentration. 10% gel was used and 30 μg sample were added to each lane. Proteins were transferred to PVDF membranes (Merck Millipore, PR05507, Carrigtwohill, Ireland) after electrophoresis. After the PVDF membrane was blocked with 5% skim milk for 60 min at 25°C, it was incubated with the primary antibody (anti-OTUD4 1:4000, anti-GAPDH 1:10000, anti-Beta Actin 1:10000, anti-MYH9 1:4000, anti-N-Cadherin 1:2000, anti-E-Cadherin 1:2000, anti-MMP9 1:3000, anti-Flag 1:1000, anti-His 1:1000) overnight at 4°C. Conjugate the corresponding primary antibody with the corresponding secondary antibody (Proteintech) (1:10000) for 60 min at room temperature. Protein bands were detected using ECL (Proteintech, PK10003) detection reagents. Signal detection was performed using the chemiluminescence gel imaging system (Tanon, 5200-Multi, Shanghai, China) The program for this system is Tanon Alldoc_x (v1.100).

### 2.4 Quantitative Real-Time PCR (qRT-PCR) and mRNA Extraction

The total mRNA extracted with TRIzol reagent was reverse transcribed. For reverse transcription, a BIOG cDNA Synthesis Kit (BioDai, 31014, Changzhou, China) was used. The reverse transcription reaction system consisted of 20 μL (DEPC water 2000/RNA concentration-7 μL, dNTP 4 μL, Primer mix 2 μL, RNA 2000/RNA concentration μL, RT Buffer 4 μL, DTT 2 μL, Hifscript 1 μL). The reverse transcription process was performed at 25°C for 5 min, 42°C for 30 min, and 85°C for 5 min. Spectrophotometer (Eppendorf, 22331, Hamburg, Germany) was used to determine RNA and cDNA concentrations. Furthermore, TOROGreen qPCR Master Mix (Transgene, AQ131-02, Beijing, China) was applied for qRT-PCR. qRT-PCR was performed with an ABI Prism 7900 system (Thermo Fisher Scientific, Waltham, MA, USA). The qPCR cycling conditions included initial denaturation (95°C for 5 min), denaturation (95°C for 10 s), and annealing/extension (60°C for 30 s). The denaturation and annealing/extension cycles were repeated 40 times. Internal control gene is GAPDH and the 2^−ΔΔCt^ method was used for relative expression differences. GAPDH-Forward: 5^′^-GGGGAGCCAAAAGGGTCATCATCT-3^′^; GAPDH-Reverse: 5^′^-GACGCCTGCTTCACCACCTTCTTG-3^′^; OTUD4-Forward: 5^′^-TTCTGATGTGGATTACAGAGGGC-3^′^; OTUD4-Reverse: 5^′^-ACGCATGTTGTCTTACTCCTGA-3^′^.

### 2.5 Cell Counting Kit-8 (CCK-8) Assay

After C4-2 and PC-3 cells (1 × 10^3^ cells per well) were cultured in 96-well plates for 1–5 days with full medium (89% base medium, 10% FBS and 1% P/S) (BasalMedia, L210KJ, Shanghai, China) (HAKATA, HB-FBS-500, Shanghai, China) (BasalMedia, S110JV, Shanghai, China) at 37°C, 10 μL CCK-8 (APExBIO, K1018, Shanghai, China) was added and incubated for 2 h at 37°C. Results were measured at 450 nm with a full wavelength scanning multifunction readout (BioTek, 800 TS, Montpelier, VT, USA). Data are displayed by Gen5 software (BioTek, Montpelier, VT, USA) (v1.11.5).

### 2.6 Colony Formation Assay

C4-2 and PC-3 cells were planted in a six-well plate at a density of 1 × 10^3^ per well with full medium (89% base medium, 10% FBS and 1% P/S) (BasalMedia, L210KJ, Shanghai, China) (HAKATA, HB-FBS-500, Shanghai, China) (BasalMedia, S110JV, Shanghai, China) at 37°C. About 14 days later, the original medium was aspirated, fixed with 4% paraformaldehyde (Solarbio, P1110, Beijing, China) for 15 min, and then stained with crystal violet (Solarbio, G1064) for 15 min. Finally, the photograph was taken (Canon, 90D, Tokyo, Japan).

### 2.7 Transwell Assay

600 μL of full medium (89% medium, 10% FBS and 1% P/S) (BasalMedia, L210KJ, Shanghai, China) (HAKATA, HB-FBS-500, Shanghai, China) (BasalMedia, S110JV, Shanghai, China) was added to the lower layer of the small chamber, and 200 μL of FBS-free medium (BasalMedia, L210KJ, Shanghai, China) was added to the upper part of the small chamber. Matrigel (MCE, HY-K6001, Shanghai, China) was coated on the membrane with 8 μm pore size. 5 × 10^4^ cells were added to the upper layer of the chamber. After 48 h of incubation with 37°C and 5% CO_2_, cells were fixed with 4% paraformaldehyde (Solarbio, Beijing, China) for 15 min and stained with crystal violet (Solarbio, G1064, Beijing, China) for 15 min. Use a cotton swab to wipe off the cells in the upper layer of the chamber. Finally, photos were taken under a microscope (Olympus, LX73, Tokyo, Japan).

### 2.8 5-Ethynyl-2^′^-deoxyuridine (EdU) Assay

First, 4 × 10^4^ cells of different groups were seeded in 24-well plates and cultured for 24 h with full medium (89% base medium, 10% FBS and 1% P/S) (BasalMedia, L210KJ, Shanghai, China) (HAKATA, HB-FBS-500, Shanghai, China) (BasalMedia, S110JV, Shanghai, China) at 37°C. EdU kit (AbbVie, KTA2031, Shanghai, China) was used for staining. Subsequently, 4% paraformaldehyde (Solarbio, P1110, Beijing, China) was used to fix the cells. After that, 1% Triton X-100 (Solarbio, T8200, Beijing, China) was used to permeabilize the cells. Finally, we stained the cell nucleus with 10 μg/mL DAPI (Solarbio, C0065, Beijing, China) for 10 min and took pictures using a fluorescence microscope (Olympus, LX73, Tokyo, Japan).

### 2.9 Immunofluorescence Assay

First, 1 × 10^4^ cells of different groups were seeded in 24-well plates and cultured for 24 h with full medium (89% base medium, 10% FBS and 1% P/S) (BasalMedia, L210KJ, Shanghai, China) (HAKATA, HB-FBS-500, Shanghai, China) (BasalMedia, S110JV, Shanghai, China) at 37°C. Then, we fixed and permeabilized the cells with 4% paraformaldehyde (Solarbio, P1110, Beijing, China) for 15 min and 1% Triton X-100 (Solarbio, T8200, Beijing, China) for 15 min. 5% BSA (Solarbio, A8020, Beijing, China) solution was used to block the cells for 30 min, and we incubated them with different primary antibodies (anti-OTUD4 1:400 and anti-MYH9 1:400) overnight at 4°C. The next day, Coralite 488 (ProteinTech, SA00013-1) or 594 (ProteinTech, SA00013-2) secondary antibodies (1:1000) were used to stain the cells for 60 min at 25°C. Finally, we stained the cell nuclei with 10 μg/mL DAPI (Solarbio, C0065) for 10 min and took pictures under a fluorescence microscope (Olympus, LX73, Tokyo, Japan).

### 2.10 Mass Spectrometry (MS) Analysis

300 μL IP lysis buffer was added to the 3 × 10^6^ cells, and the cells were suspended. The IP lysis buffer was reacted at 4°C for 30 min. 4 μg OTUD4 primary antibody (anti-OTUD4 or anti-MYH9) was added to protein lysis buffer and incubated overnight at 4°C with rotation. The next day, agarose beads were added to the protein lysis buffer and incubated at 4°C for 4 h. A filter column was added to the protein lysis buffer and centrifuged at 11,000× *g* for 5 min at 4°C. Finally, the eluent was added to the filter column and reacted at room temperature for 5 min. All of the above processes used the immunoprecipitation kit (Proteintech, PK10007). The samples immunoprecipitated with OTUD4 antibody were subjected to gel electrophoresis (10% gel). Afterwards, the gel was placed in 500 mL of Coomassie Brilliant Blue solution and shaken at 37°C for 2 h. The Coomassie Brilliant Blue-stained and excised bands were destained in a solution containing 100 mM ammonium bicarbonate and acetonitrile and left overnight. Subsequent processing and data analysis were performed by Personalbio (Shanghai, China). The database used in this study was GCF_000001405.39_GRCh38.p13_protein.FASTA. Data were analyzed using Thermo Scientific Proteome Discoverer (v3.1, Thermo Fisher Scientific, Waltham, MA, USA).

### 2.11 Immunohistochemical (IHC) Analysis

All tissue sections were obtained from the Department of Pathology, The Second Hospital of Tianjin Medical University. This study was approved by the Ethics Committee of the Second Hospital of Tianjin Medical University (approval No. KS2024029). This study complies with the Declaration of Helsinki. Informed consent was obtained from all patients whose tissues were used. The tissue sections were 4 μm thick. All tissue sections were embedded in paraffin at the Department of Pathology, The Second Hospital of Tianjin Medical University. IHC staining was performed using an IHC kit (ProteinTech, PK10017) according to the instructions. The slides underwent dewaxing, rehydration, antigen retrieval, and endogenous peroxidase and microbial inactivation processes sequentially. After mounting with goat serum, the slides were incubated overnight at 4°C with antibodies (1:400), followed by the addition of a reaction enhancer and an enhanced enzyme-labeled goat anti-rabbit/mouse IgG polymer. The slides were washed with PBS between each step. The detection signal was enhanced using the DAB, counterstained with hematoxylin, and then dehydrated and mounted. The main intensity of tumor cell staining in each section was (1: weak; 2: medium; 3: strong). Finally, photos were taken using an optical microscope (Olympus, LX73, Tokyo, Japan).

### 2.12 Hematoxylin-Eosin (HE) Staining Analysis

All tissue sections were obtained from the Department of Pathology, The Second Hospital of Tianjin Medical University. This study was approved by the Ethics Committee of the Second Hospital of Tianjin Medical University (approval No. KS2024029). This study complies with the Declaration of Helsinki. Informed consent was obtained from all patients whose tissues were used. The tissue sections were 4 μm thick. All tissue sections were embedded in paraffin at the Department of Pathology, The Second Hospital of Tianjin Medical University. HE staining was performed by the HE staining kit (Solarbio, G1120, Beijing, China). After dewaxing and hydrating the sections, hematoxylin was added to cover the sections for 5 min at room temperature. After washing the sections with PBS, add eosin to cover the sections for 2 min at room temperature. After dehydration, mount the sections with neutral resin. Finally, photos were taken using an optical microscope (Olympus, LX73, Tokyo, Japan).

### 2.13 Tumor Xenografts in Nude Mice

All animal experiments were approved by the Ethical Committee of the Tianjin Medical University (approval No. 2023006) and all methods were performed in accordance with the regulations of the Ethical Committee of the Tianjin Medical University and ARRIVE guidelines. 12 male BALB/c nude mice (Yishengyuan, Tianjin, China), each weighing approximately 15 g, were housed in an Association for Assessment and Accreditation of Laboratory Animal Care International (AAALAC) approved facility in Tianjin Medical University, under controlled temperature (24°C) and a 12 h light/dark cycle. Four-week-old nude mice were randomly divided into two groups (EV group and OTUD4 group). Cells from different groups were injected (each nude mouse was injected with 2 × 10^6^ cells) subcutaneously into the groin area of nude mice. After the tumors began to grow for 14 days (the largest tumor approached but did not exceed 2 cm in diameter), the nude mice were sacrificed with the cervical dislocation method, and the tumors were removed. The tumors volume (calculated as 0.5 × length × width^2^) and weight was measured.

### 2.14 Bioinformatics

We obtained the gene expression data from 502 prostate adenocarcinoma (PRAD) tumor tissues and 52 normal tissues in The Cancer Genome Atlas (TCGA) from the University of California, Santa Cruz Genome Browser (UCSC Xena) website (https://xena.ucsc.edu/), while the expression data of normal tissues from Genotype Tissue Expression (GTEx) were also utilized to increase the sample size of the normal group. The “ggplot2” package (v4.0.0) and the “survival” package (v3.5.8) were used to plot box plots and survival curves on the R platform (v4.3.3), respectively. To explore the biological functions associated with MYH9, we performed differential expression analysis on MYH9 using the “limma” package (v3.58.1), and multiple testing correction was applied, and an FDR < 0.05 was considered statistically significant. Subsequently, we conducted Kyoto Encyclopedia of Genes and Genomes (KEGG) and Gene Ontology (GO) analysis based on the MYH9 differentially expressed gene sets obtained above using the “clusterProfiler” package (v4.10.1), and q-value < 0.05 was considered statistically significant.

### 2.15 Statistical Analysis

The experiment data were analyzed via GraphPad Prism (v10.6, GraphPad Software, San Diego, CA, USA). All experiment data are presented by mean ± standard deviation (SD). *t*-test was used to compare the differences between two groups. Comparisons of more than two groups were performed using one-way ANOVA analysis. All *t*-tests are unpaired *t*-tests and all *t*-tests are two-tailed. *p* < 0.05 was considered to be statistically significant (*p < 0.05, **p < 0.01, ***p < 0.001).

## 3 Results

### 3.1 OTUD4 Was Significantly Reduced in Prostate Cancer Tissue

**Figure 1:**
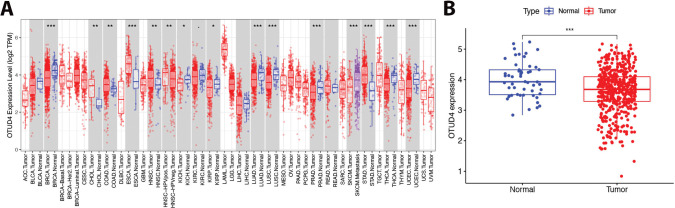

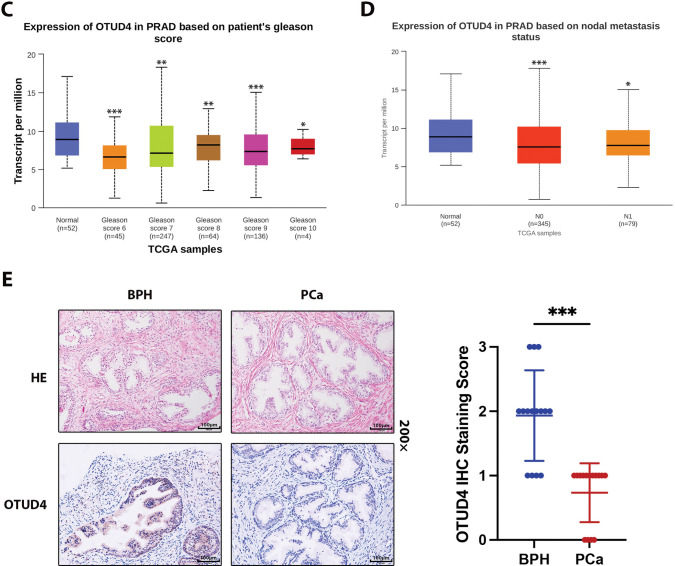
Differential expression of Ovarian Tumor Family Deubiquitinase 4 (OTUD4) in normal tissues and tumor tissues. (**A**) The expression differences of OTUD4 in normal tissues and tumor tissues in each system. (**B**) The difference in OTUD4 expression between normal prostate tissue and prostate cancer was analyzed using the The Cancer Genome Atlas (TCGA) database. (**C**) Expression of OTUD4 in prostatic adenocarcinoma (PRAD) based on patient’s gleason scores. (**D**) Expression of OTUD4 in PRAD based on nodal metastasis status. (**E**) Immunohistochemical (HE) and Hematoxylin-Eosin (IHC) immunohistochemistry of benign prostatic hyperplasia and prostate cancer. All data were expressed as the mean ± Standard Deviation (SD), n = 15. Student’s *t*-test was performed to analyze significance between two groups. All *t*-tests are unpaired *t*-tests and all *t*-tests are two-tailed. Comparisons of more than two groups were performed using one-way ANOVA analysis. *p < 0.05, **p < 0.01, ***p < 0.001

In order to explore the expression difference of OTUD4 in normal tissues and tumor tissues, bioinformatics methods were used to analyze the TCGA database. We found that OTUD4 was expressed at low levels in a variety of tumors, especially in urinary system tumors (Fig. 1A). Importantly, OTUD4 was significantly reduced in prostate cancer compared to normal prostate (Fig. 1B). Although there was no obvious relationship between OTUD4 expression and Gleason score, OTUD4 expression was generally decreased in prostate cancer (Fig. 1C). Similarly, there was no obvious relationship between OTUD4 and the degree of lymph node metastasis (Fig. 1D). Subsequently, we collected 15 pairs of tissue samples of benign prostatic hyperplasia (BPH) and prostate cancer for immunohistochemical staining in our center. The results were consistent with the above bioinformatics results, namely that OTUD4 was significantly reduced in prostate cancer (Fig. 1E). Taken together, these results indicate that OTUD4 expression is significantly reduced in prostate cancer tissues. Based on this, we speculate that OTUD4 may play the role of a tumor suppressor gene in prostate cancer.

### 3.2 OTUD4 Inhibits the Proliferation and Invasion of Prostate Cancer Cells

**Figure 2:**
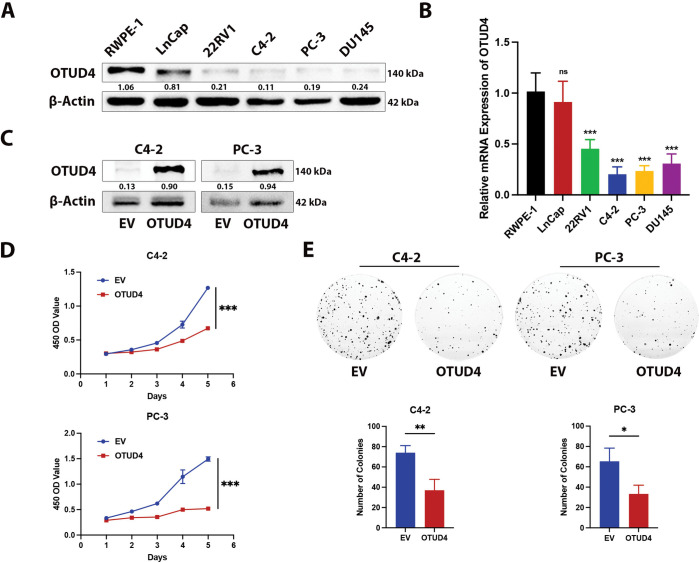

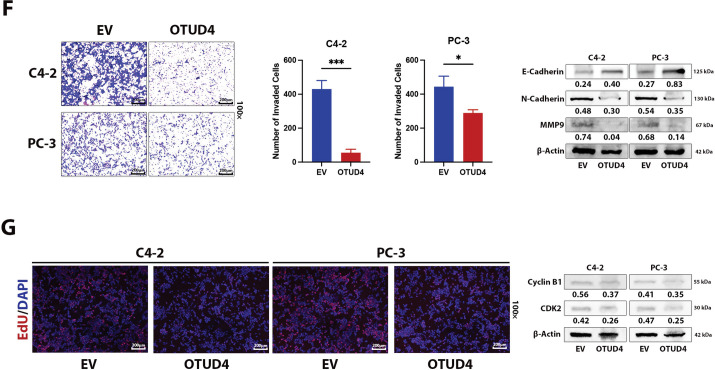
Effect of OTUD4 on the biological behavior of prostate cancer. (**A**) Expression levels of OTUD4 protein in five prostate cancer cell lines. (**B**) OTUD4 mRNA levels in five prostate cancer cell lines. (**C**) Overexpression of OTUD4. (**D**) Effect of OTUD4 on the viability of prostate cancer cells. (**E**) Effect of OTUD4 on the proliferation ability of prostate cancer cells. (**F**) Effect of OTUD4 on the invasion ability of prostate cancer cells. (**G**) Effect of OTUD4 on mitosis in prostate cancer. All data were expressed as the mean ± SD, n = 3. Student’s *t*-test was performed to analyze significance between two groups. All *t*-tests are unpaired *t*-tests and all *t*-tests are two-tailed. Comparisons of more than two groups were performed using one-way ANOVA analysis. *p < 0.05, **p < 0.01, ***p < 0.001, ns: no significance

**Figure 3:**
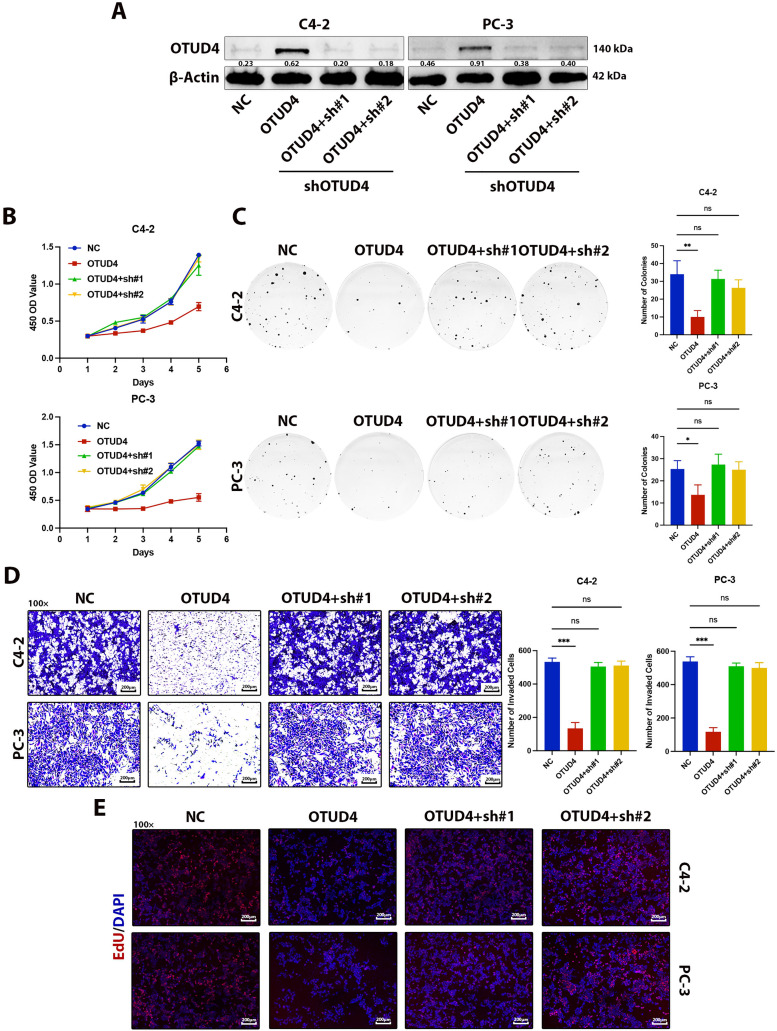
Effects of OTUD4 overexpression and knockdown on the biological behavior of prostate cancer cells. (**A**) Western Blot assay was used to detect changes in OTUD4 levels. (**B**) Effect of OTUD4 on the activity of prostate cancer cells. (**C**) Effect of OTUD4 on the proliferation ability of prostate cancer cells. (**D**) Effect of OTUD4 on the invasion ability of prostate cancer cells. (**E**) Effect of OTUD4 on mitosis of prostate cancer cells. All data were expressed as the mean ± SD, n = 3. Comparisons of more than two groups were performed using one-way ANOVA analysis. *p < 0.05, **p < 0.01, ***p < 0.001, ns: no significance

To further explore how OTUD4 can affect the biological behaviors of prostate cancer cells, such as proliferation and invasion, we first detected the protein and mRNA levels of OTUD4 in RWPE-1 (normal prostate cells), LnCap, 22RV1, C4-2, PC-3 and DU145. The results showed that the expression levels of OTUD4 were lowest in C4-2 and PC-3 cell lines, and the expression of OTUD4 in RWPE-1 was significantly higher than that in prostate cancer cell lines, both at the protein and mRNA levels (Fig. 2A,B). The above results in cells are consistent with those in tissues. Subsequently, we constructed OTUD4 overexpression cell models in C4-2 and PC-3 cell lines for subsequent experiments (Fig. 2C). According to the results of CCK-8 experiments, high expression of OTUD4 can inhibit the activity of prostate cancer cells (Fig. 2D). In addition, OTUD4 was able to inhibit the proliferation ability of prostate cancer through plate cloning assay (Fig. 2E). Importantly, OTUD4 could significantly inhibit the invasion ability of prostate cancer cells through Transwell experiments, and the results of Western Blot experiments showed that key indicators related to invasion (E-Cadherin, N-Cadherin and MMP9) had corresponding changes (Fig. 2F). Finally, we found through EdU experiments that OTUD4 can effectively inhibit the mitotic ability of prostate cancer cells. Similarly, Western Blot results showed that key proteins in the cell cycle (CDK2 and Cyclin B1) were reduced in prostate cancer cells overexpressing OTUD4 (Fig. 2G). In addition, to ensure the reliability of the above results, we knocked down the cell line overexpressing OTUD4. Subsequently, the same experimental method was used for verification. The results showed that when OTUD4 was reduced, the proliferation and invasion ability of cells were significantly reduced. However, when OTUD4 was reduced, the biological behavior of cells was reversed (Fig. 3A–E). Overall, these results indicate that OTUD4 can inhibit the biological behavior of prostate cancer cell lines and suggest that OTUD4 plays the role of a tumor suppressor gene in prostate cancer.

### 3.3 OTUD4 Interacts with Downstream Target Protein MYH9 and Inhibits Its Degradation

**Figure 4:**
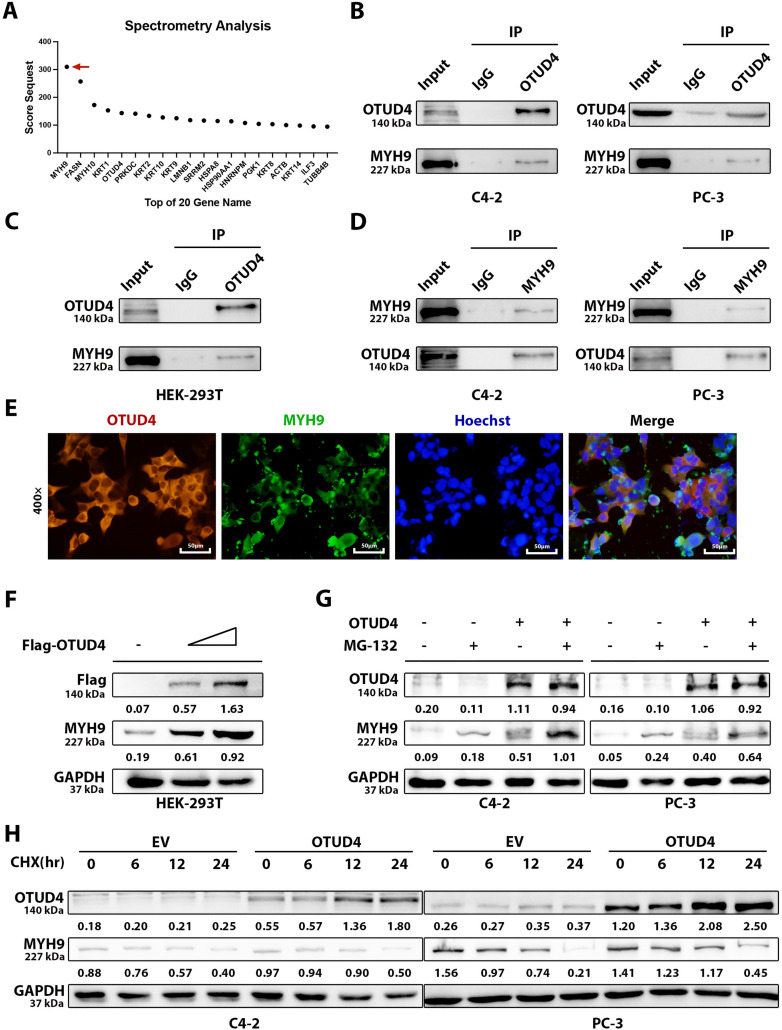
OTUD4 interacts with myosin heavy chain 9 (MYH9). (**A**) Mass spectrometry analysis of IP products of OTUD4. The red arrow points to MYH9, which has the highest rating. (**B**) Co-IP analysis of the interaction between OTUD4 and MYH9. (**C**) Co-IP analysis of OTUD4 interaction with MYH9 in HEK-293T cell line. (**D**) Verification that MYH9 can interact with OTUD4. (**E**) Verification of the subcellular localization of OTUD4 and MYH9. (**F**) The relationship between the expression levels of OTUD4 and MYH9 was verified in a dose-dependent manner. (**G**) Verification of MYH9 degradation via the proteasome pathway by Z-Leo-Leo-Leo-al (MG132). (**H**) Verification that OTUD4 can inhibit MYH9 protein degradation in a time-dependent manner

To explore how OTUD4 inhibits prostate cancer, we then performed protein mass spectrometry analysis on the IP products of OTUD4. We ranked the proteins that were found to interact with OTUD4 according to the degree of association, and the result showed that MYH9 had the highest score (Fig. 4A). To verify the accuracy of the mass spectrometry results, we performed Co-IP experiments on C4-2 and PC-3 cell lines. We found that MYH9 could be detected in the IP product of OTUD4 (Fig. 4B). In addition, since HEK-293T cells were needed for subsequent experiments, we also performed the above analysis on HEK-293T cells, and the results were consistent (Fig. 4C). Similarly, OTUD4 was detected when the IP product of MYH9 was examined by Co-IP assay in C4-2 and PC-3 cell lines (Fig. 4D). Next, to further verify the interaction between OTUD4 and MYH9, we performed immunofluorescence experiments. The results showed that OTUD4 and MYH9 have the same subcellular localization, that is, they are both located in the cytoplasm (Fig. 4E). Since OTUD4 is a deubiquitinating-related protein, as the expression level of OTUD4 increases, the protein expression level of MYH9 also increases, showing a dose-dependency (Fig. 4F). Next, to verify the deubiquitination effect of OTUD4 on MYH9, Z-Leo-Leo-Leo-al (MG132) (an inhibitor of the ubiquitin-proteasome pathway) was used. MG132 significantly inhibited the degradation of MYH9, especially when OTUD4 was overexpressed, indicating that OTUD4 protects MYH9 from degradation by inhibiting the ubiquitin-proteasome pathway (Fig. 4G). Finally, we used Cycloheximide (CHX) (a protein synthesis inhibitor) to treat cells in the control group and OTUD4 overexpression group. Although the expression of MYH9 decreased with the extension of CHX treatment time, the expression level of MYH9 was significantly higher than that of the control group when OTUD4 was overexpressed, which indicated the protective effect of OTUD4 on MYH9 protein (Fig. 4H). In summary, we found that on the one hand, OTUD4 can interact with downstream MYH9, and on the other hand, OTUD4 can protect MYH9 from degradation by inhibiting the ubiquitin-proteasome pathway.

### 3.4 OTUD4 Protects MYH9 from Degradation through Deubiquitination

**Figure 5:**
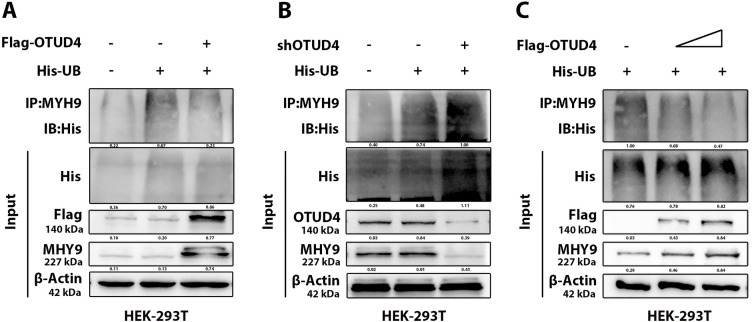
Ubiquitination validation. (**A**) Effect of overexpression of OTUD4 on MYH9 ubiquitination. (**B**) Effect of OTUD4 knockdown on MYH9 ubiquitination. (**C**) Overexpression of OTUD4 affected MYH9 ubiquitination in a dose-dependent manner

In order to further explore the possibility of OTUD4 deubiquitinating MYH9, we conducted ubiquitination-related experiments. As shown in the figure, MYH9 expression decreased when UB was overexpressed. However, when OTUD4 was overexpressed, MYH9 expression increased and the level of UB associated with MYH9 also decreased (Fig. 5A). Conversely, when OTUD4 was knocked down, MYH9 expression was decreased and the level of MYH9-associated UB was increased (Fig. 5B). Finally, in a dose-dependent manner, we found that as OTUD4 expression increased, MYH9 expression increased and MYH9-related UB decreased (Fig. 5C). In conclusion, based on the above results, it was confirmed that OTUD4 protected MYH9 through deubiquitination.

### 3.5 MYH9 Significantly Inhibits Prostate Cancer through Interactions with Cell Adhesion Molecules

**Figure 6:**
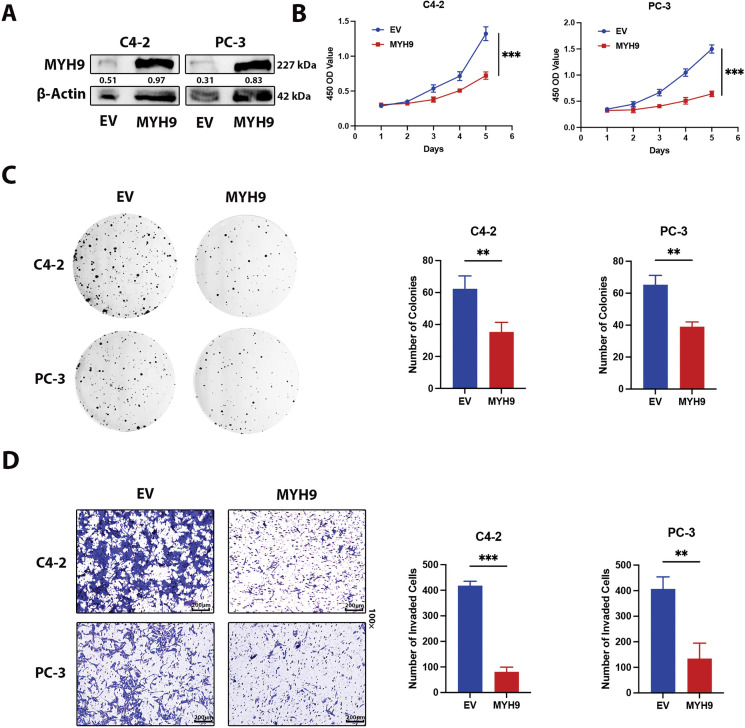

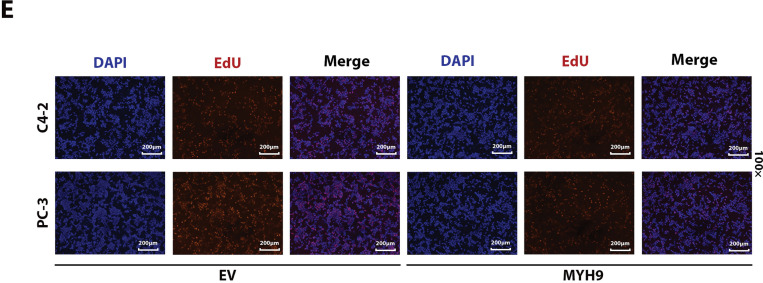
Effect of MYH9 overexpression on the biological behavior of prostate cancer. (**A**) Western blot analysis to verify the efficiency of MYH9 overexpression. (**B**) CCK-8 assay to verify the effect of MYH9 overexpression on cell viability. (**C**) Clonogenic assay to verify the effect of MYH9 overexpression on cell proliferation. (**D**) Transwell assay to verify the effect of MYH9 on cell invasion. (**E**) Effect of OTUD4 on mitosis in prostate cancer. All data were expressed as the mean ± SD, n = 3. Student’s *t*-test was performed to analyze significance between two groups. All *t*-tests are unpaired *t*-tests and all *t*-tests are two-tailed. **p < 0.01, ***p < 0.001

Previous studies have shown that OTUD4 expression is reduced in prostate cancer and that it functions as a tumor suppressor gene. Importantly, we have demonstrated through mass spectrometry analysis and Co-IP experiments that OTUD4 inhibits MYH9 protein degradation. To further investigate whether MYH9 plays a tumor suppressive role in prostate cancer, we conducted the following work. First, the MYH9 overexpression efficiency was verified by Western Blot (Fig. 6A). When MYH9 was elevated, prostate cancer cell activity was significantly inhibited as measured by CCK-8 assay (Fig. 6B). MYH9 significantly inhibited the proliferation ability of prostate cancer through clonogenic assays (Fig. 6C). The invasive ability of prostate cells was also inhibited when MYH9 was highly expressed (Fig. 6D). Finally, the results of the EdU experiment demonstrated that MYH9 could inhibit mitosis in prostate cancer (Fig. 6E). In addition, we conducted a series of bioinformatics analyses on MYH9 (Fig. S1A–D). The results showed that MYH9 expression was significantly decreased in prostate cancer tissue compared to normal prostate tissue. On the other hand, patients with prostate cancer with elevated MYH9 expression had a higher expected survival rate than those with low MYH9 expression, although the differences in survival analysis were not significant. Importantly, GO and KEGG enrichment analyses revealed that MYH9 was highly associated with cell adhesion and cell adhesion molecules. Cell adhesion molecules are members of the immunoglobulin superfamily. Previous studies have shown that they can inhibit the development of non-small cell lung cancer and have been identified as important tumor suppressor genes. Importantly, cell adhesion molecules are not expressed in prostate, breast, and lung cancers. Furthermore, cell adhesion molecules can act as tumor antigens to activate NK cells and CD8^+^ T cells, exerting anti-tumor effects [25]. Thus far, we have obtained conclusive evidence that OTUD4 acts as a tumor suppressor gene and is down expressed in prostate cancer, and that OTUD4 reduces the ubiquitination and degradation of MYH9, another tumor suppressor gene. MYH9 interacts with cell adhesion molecules to inhibit the occurrence and development of prostate cancer.

### 3.6 OTUD4 Inhibits Prostate Cancer Xenograft Tumor Growth

**Figure 7:**
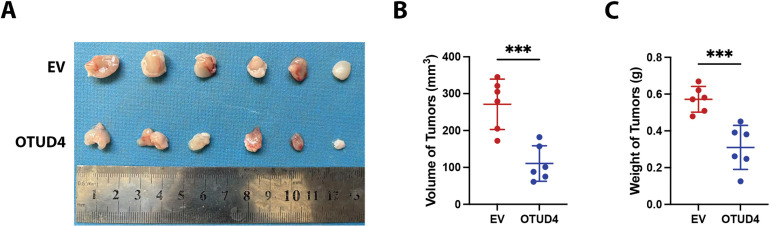

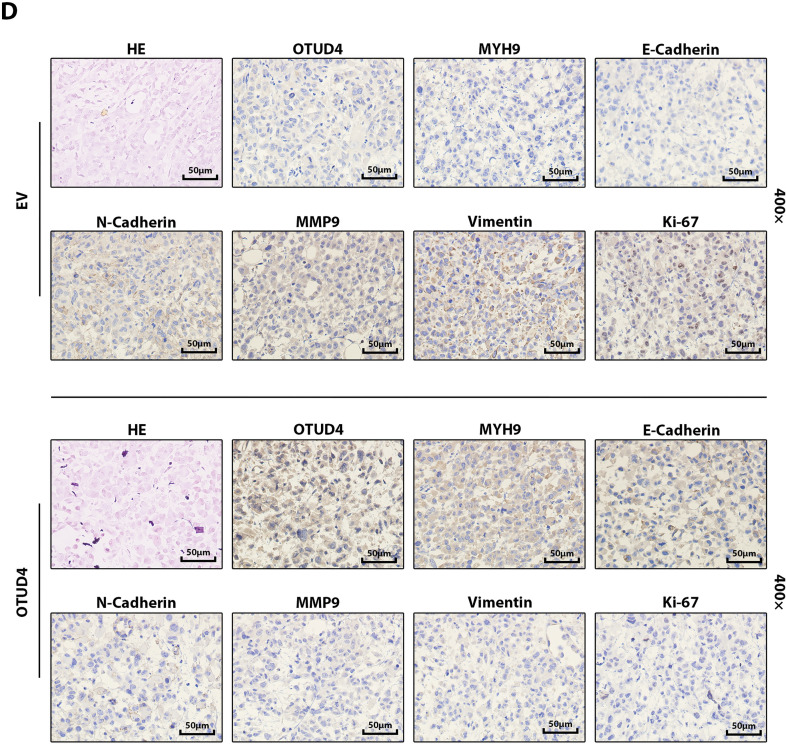
Effects of OTUD4 on tumor growth *in vivo*. (**A**) Photo of the two groups of tumors. (**B**) Statistical graph of tumor volume (calculated as 0.5 × length × width^2^) in the two groups. (**C**) Statistical graph of tumor weights in the two groups. (**D**) IHC and HE staining of tumor-related indicators in the two groups. All data were expressed as the mean ± SD, n = 6. Student’s *t*-test was performed to analyze significance between two groups. All *t*-tests are unpaired *t*-tests and all *t*-tests are two-tailed. ***p < 0.001

The above experiments fully prove that OTUD4 can effectively inhibit the proliferation of prostate cancer *in vitro*. Here, to verify that OTUD4 can inhibit prostate cancer *in vivo*, we implanted control PC-3 cells and PC-3 cells overexpressing OTUD4 into the subcutaneous tissues of nude mice. By comparing the dissected tumors, we found that the volume (calculated as 0.5 × length × width^2^) and weight of the tumors in the control group were higher than those in the OTUD4 overexpression group (Fig. 7A–C). After paraffin embedding and sectioning of the tumor tissue, we performed HE staining and IHC staining. The results were consistent with the *in vitro* experiment, that is, as the expression of OTUD4 increased, MYH9 also increased, but the invasion ability and activity were inhibited (Fig. 7D). In summary, on the one hand, our results show that OTUD4 can inhibit prostate cancer cells and OTUD4 can prevent the ubiquitination and degradation of the tumor suppressor gene MYH9 *in vitro*. On the other hand, we verified that OTUD4 can also play a tumor suppressive role in prostate cancer tissues *in vivo*.

## 4 Discussion

Prostate cancer ranks second among cancer-related deaths in men worldwide [1]. Therefore, it is urgent to find new drug targets and new diagnostic indicators.

OTUD4 is a deubiquitinating enzyme belonging to the ovarian tumor-associated protease domain protein family. Studies have shown that OTUD4 is a potential predictor of several human cancers [10]. It is involved in cleaving K48- and K63-linked polyubiquitin chains and participates in DNA alkylation damage repair, which is important in cancer radiotherapy and chemotherapy [10,14,26–28]. Integrating previous reports, we found that OTUD4 expression varies across different tumor types. Specifically, OTUD4 levels are elevated in pancreatic adenocarcinoma, stomach adenocarcinoma, thymoma, glioma, and triple-negative breast cancer [10,11,29]. In contrast, OTUD4 expression is significantly decreased in bladder urothelial carcinoma, breast invasive carcinoma, liver hepatocellular carcinoma, lung adenocarcinoma, lung squamous cell carcinoma, ovarian serous cystadenocarcinoma, prostate adenocarcinoma, skin cutaneous melanoma, testicular germ cell tumors, thyroid carcinoma, and uterine corpus endometrial carcinoma [10]. These results suggest that OTUD4 plays distinct roles in different tumor types. Consistent with our expectation and previous literature, OTUD4 promotes glioma progression by deubiquitinating CDK1 and affecting the MAPK pathway [11]. OTUD4 also regulates glycolysis to promote pancreatic tumor progression [13]. However, other studies indicate that OTUD4 can suppress other tumors. For example, OTUD4 regulates the ATM/CHK2/P53 pathway to inhibit non-small cell lung cancer (NSCLC) [14]. Importantly, ubiquitination plays diverse roles in prostate cancer. Previous studies have shown that ubiquitin-specific peptidase 9, X-linked (USP9X), inhibits the degradation of AlkB homolog 5 (ALKBH5) via deubiquitination, thereby enhancing prostate cancer resistance to alkylating damage [30]. Conversely, another report demonstrated that inactivation of ubiquitin domain-containing protein 1 (UBTD1) leads to overactivation of Ras homolog family member A (RhoA), enhancing the invasive ability of prostate cancer [31]. Collectively, these findings indicate that OTUD4 can deubiquitinate different substrates to exert either promotive or inhibitory effects on tumor progression depending on the cancer type. This prompted our interest in the role of OTUD4 in prostate cancer. Our results clearly demonstrate that OTUD4 significantly inhibits prostate cancer progression both *in vitro* and *in vivo*. More importantly, mass spectrometry analysis revealed that OTUD4 interacts with MYH9 in prostate cancer and inhibits MYH9 degradation via deubiquitination.

MYH9 encodes the heavy chain of non-muscle myosin IIA (NMIIA). Previous studies have shown that mutations or abnormal expression of MYH9 are associated with tumorigenesis [20]. For instance, the tumor suppressor function of p53 depends on MYH9 activity in head and neck cancer [23]. In this study, we confirmed that OTUD4 effectively inhibits prostate cancer growth *in vitro* and *in vivo*. To explore the mechanism of OTUD4-mediated tumor suppression, we performed mass spectrometry on the OTUD4 immunoprecipitates and found that OTUD4 interacts with MYH9. Furthermore, our results indicate that OTUD4 does not alter the subcellular localization of MYH9. The MYH9 antibody used in this study specifically recognizes NMIIA. Consistent with previous reports, MYH9 functions as a tumor suppressor. We further determined that OTUD4 inhibits MYH9 degradation through deubiquitination, thereby suppressing tumor growth. To confirm the effect of MYH9 on prostate cancer, we overexpressed MYH9 in prostate cancer cells and found that MYH9 significantly inhibit the activity, proliferation and invasion of prostate cancer cell lines. Importantly, our work demonstrates that MYH9 interacts with downstream cell adhesion molecules, which are known key tumor suppressors in various cancers. This result further supports the tumor-inhibitory role of MYH9 in prostate cancer. In previous glioblastoma studies, OTUD4 was shown to stabilize CDK1 by removing K11-, K29-, and K33-linked polyubiquitination, thereby affecting the downstream MAPK pathway and promoting glioblastoma progression-leading to the proposal of the OTUD4-CDK1-MAPK axis in glioblastoma [11]. Additionally, OTUD4 enhances glycolysis and promotes pancreatic ductal adenocarcinoma (PDAC) progression by stabilizing sodium/glucose cotransporter 2 (SGLT2) via deubiquitination [13]. In contrast, OTUD4 enhances radiosensitivity in NSCLC by interacting with the ATM/CHK2/P53 signaling pathway and inhibiting homologous recombination repair of ionizing radiation-induced DNA double-strand breaks [14]. In summary, OTUD4 can either promote or suppress tumors in different contexts by stabilizing specific target proteins and modulating distinct signaling pathways. Therefore, the research value of OTUD4 is highly promising across different tumor types.

Overall, our results demonstrate that OTUD4 expression is reduced in prostate cancer. Using an OTUD4-overexpressing cell line model, we confirmed that OTUD4 inhibits prostate cancer progression both *in vitro* and *in vivo*. Importantly, our mechanistic analysis revealed that OTUD4 interacts with MYH9 and inhibits its degradation via deubiquitination in prostate cancer. Consistent with previous studies, our findings indicate that MYH9 inhibits the viability, proliferation, and invasion of prostate cancer cell lines. Furthermore, we show that MYH9 influences cell adhesion molecules, thereby suppressing prostate cancer. These findings fill a gap in OTUD4 research in prostate cancer and elucidate its mechanism of action: OTUD4 inhibits prostate cancer progression by stabilizing MYH9 and subsequently affecting cell adhesion molecules. Therefore, OTUD4 holds great promise as a prognostic marker for prostate cancer and as a target for future therapeutic strategies.

Although this study revealed the role of OTUD4 and its downstream target MYH9 in prostate cancer, there are still limitations in this study. First, the ubiquitination sites affected by OTUD4 were not observed in this study. Second, we did not conduct validation of the relevant mechanisms *in vivo*. Therefore, these shortcomings should become the direction and focus of our subsequent research.

## Supplementary Materials



## Data Availability

The data that support the findings of this study are available from the Corresponding Author HW upon reasonable request.

## Supplementary Materials

The supplementary material is available online at https://www.techscience.com/doi/10.32604/or.2025.073455/s1.
