# Supplementary material for: OTUD4 Inhibits Prostate Cancer by Deubiquitinating MYH9
Source: Oncol Res. 2026 Mar 23;34(4):32. doi: 10.32604/or.2025.073455 (PMC13040314; doi:10.32604/or.2025.073455)
Supplement: Supplementary file 1 [file OncolRes-34-73455-s001.docx]

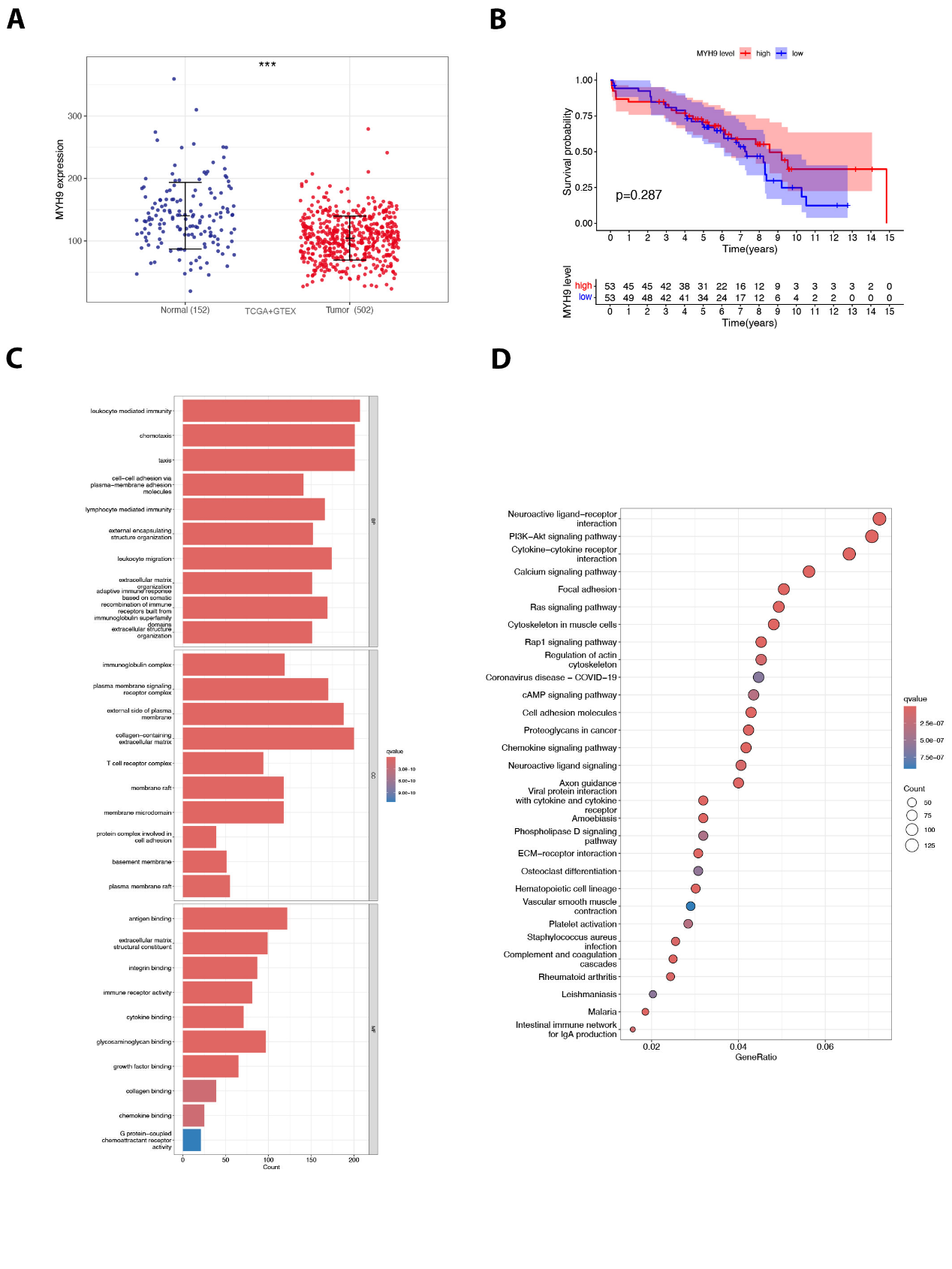


**Figure S1.** Bioinformatics analysis of myosin-9 (MYH9). (A) Differential expression analysis of MYH9 using the The Cancer Genome Atlas (TCGA) and Genotype Tissue Expression (GTE) databases. (B) Survival analysis of MYH9 differential expression. (C) Gene Ontology (GO) enrichment analysis. (D) Kyoto Encyclopedia of Genes and Genomes (KEGG) enrichment analysis. All data were expressed as the mean ± Standard Deviation (SD). Student’s T-test was performed to analyze significance between two gruops. All T-tests are unpaired T-tests and all T-tests were two-tailed. ****P* < 0.001.
